# 1-(1-Hy­droxy-8-methyl-9*H*-carbazol-2-yl)ethanone

**DOI:** 10.1107/S1600536810045769

**Published:** 2010-11-13

**Authors:** R. Archana, K. Prabakaran, K. J. Rajendra Prasad, A. Thiruvalluvar, R. J. Butcher

**Affiliations:** a PG Research Department of Physics, Rajah Serfoji Government College (Autonomous), Thanjavur 613 005, Tamilnadu, India; b Department of Chemistry, Bharathiar University, Coimbatore 641 046, Tamilnadu, India; c Department of Chemistry, Howard University, 525 College Street NW, Washington, DC 20059, USA

## Abstract

The title compound, C_15_H_13_NO_2_, crystallizes with four independent mol­ecules (*A*, *B*, *C* and *D*) in the asymmetric unit. The carbazole units are almost planar [maximum deviations = 0.015 (3) for *A*, 0.024 (3) for *B*, 0.026 (3) for *C* and 0.046 (3) Å for *D*]. In all four mol­ecules, there is an O—H⋯O hydrogen bond involving the hy­droxy substituent and the carbonyl O atom of the adjacent acetyl group, which forms a six-membered ring. In the crystal, the four independent mol­ecules are linked *via* N—H⋯O and C—H⋯O inter­actions.

## Related literature

For the biological and pharmacological activity of carbazole alkaloids, see: Hagiwara *et al.* (2000 ▶); Randelia & Patel (1982 ▶); Tovey *et al.* (1998 ▶). For the synthesis of various hetero-annulated carbazole derivatives, see: Vandana & Prasad (2004 ▶); Vandana *et al.* (2004 ▶).
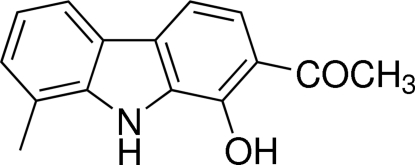


## Experimental

### 

#### Crystal data

C_15_H_13_NO_2_
*M* *_r_* = 239.26
Monoclinic, 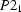

*a* = 7.4602 (2) Å
*b* = 12.7353 (3) Å
*c* = 24.9325 (5) Å
β = 92.319 (2)°
*V* = 2366.84 (10) Å^3^
*Z* = 8
Cu *K*α radiation
μ = 0.72 mm^−1^
*T* = 295 K
0.47 × 0.31 × 0.11 mm

#### Data collection

Oxford Diffraction Xcalibur Ruby Gemini diffractometer
Absorption correction: multi-scan (*CrysAlis PRO*; Oxford Diffraction, 2010 ▶) *T*_min_ = 0.746, *T*_max_ = 1.000
4770 measured reflections
4770 independent reflections
4075 reflections with *I* > 2σ(*I*)

#### Refinement

*R*[*F* ^2^ > 2σ(*F* ^2^)] = 0.042
*wR*(*F* ^2^) = 0.122
*S* = 1.05
4770 reflections
685 parameters
5 restraints
H atoms treated by a mixture of independent and constrained refinement
Δρ_max_ = 0.16 e Å^−3^
Δρ_min_ = −0.18 e Å^−3^

### 

Data collection: *CrysAlis PRO* (Oxford Diffraction, 2010 ▶); cell refinement: *CrysAlis PRO*; data reduction: *CrysAlis PRO*; program(s) used to solve structure: *SHELXS97* (Sheldrick, 2008 ▶); program(s) used to refine structure: *SHELXL97* (Sheldrick, 2008 ▶); molecular graphics: *ORTEP-3* (Farrugia, 1997 ▶) and *PLATON* (Spek, 2009 ▶); software used to prepare material for publication: *PLATON*.

## Supplementary Material

Crystal structure: contains datablocks global, I. DOI: 10.1107/S1600536810045769/hg2736sup1.cif

Structure factors: contains datablocks I. DOI: 10.1107/S1600536810045769/hg2736Isup2.hkl

Additional supplementary materials:  crystallographic information; 3D view; checkCIF report

## Figures and Tables

**Table 1:** Hydrogen-bond geometry (Å, °)

*D*—H⋯*A*	*D*—H	H⋯*A*	*D*⋯*A*	*D*—H⋯*A*
O1*A*—H1*A*⋯O14*A*	0.85 (4)	1.80 (4)	2.563 (3)	147 (5)
O1*B*—H1*B*⋯O14*B*	0.83 (4)	1.85 (4)	2.574 (4)	145 (5)
O1*C*—H1*C*⋯O14*C*	0.84 (3)	1.79 (3)	2.575 (4)	156 (5)
O1*D*—H1*D*⋯O14*D*	0.85 (4)	1.87 (4)	2.573 (4)	139 (5)
N12*A*—H12*A*⋯O14*B*^i^	0.79 (5)	2.27 (5)	3.052 (4)	172 (5)
N12*B*—H12*B*⋯O14*A*^i^	0.93 (5)	2.33 (5)	3.176 (4)	153 (5)
N12*C*—H12*C*⋯O14*D*^ii^	0.88 (4)	2.28 (4)	3.133 (4)	163 (3)
N12*D*—H12*D*⋯O14*C*^ii^	0.86 (4)	2.26 (4)	3.105 (4)	168 (3)
C15*A*—H15*A*⋯O1*B*^iii^	0.96	2.47	3.422 (5)	173
C15*B*—H15*D*⋯O1*A*^iii^	0.96	2.60	3.433 (5)	145
C15*C*—H15*I*⋯O1*D*^iv^	0.96	2.53	3.445 (5)	160
C15*D*—H15*J*⋯O1*C*^iv^	0.96	2.44	3.388 (5)	170

Symmetry codes: (i) 
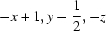
; (ii) 
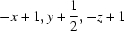
; (iii) 
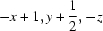
; (iv) 
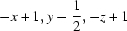
.

**Fig. 1.**
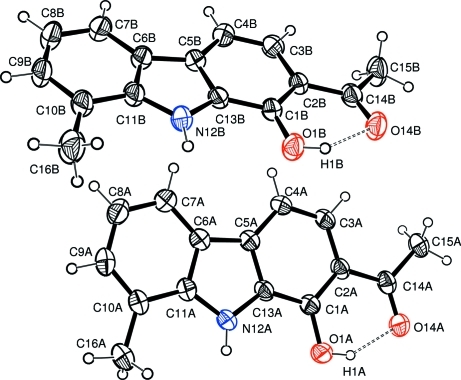
A view of the molecular structure of the two independent molecules (A and B), showing the atom-numbering scheme and displacement ellipsoids drawn at the 30% probability level. H atoms are shown as small spheres of arbitrary radius and the intramolecular O—H···O hydrogen bonds as dashed lines.

**Fig. 2.**
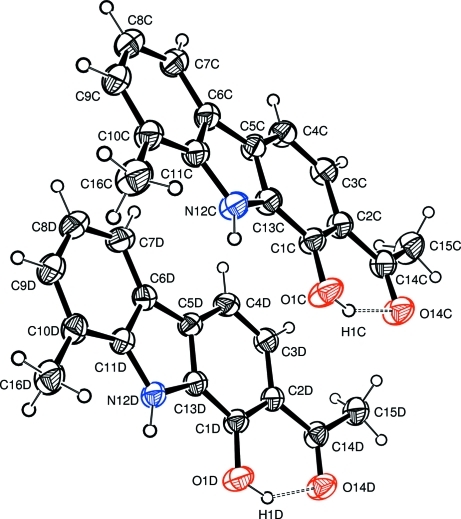
A view of the molecular structure of the two independent molecules (C and D), showing the atom-numbering scheme and displacement ellipsoids drawn at the 30% probability level. H atoms are shown as small spheres of arbitrary radius and the intramolecular O—H···O hydrogen bonds as dashed lines.

**Fig. 3.**
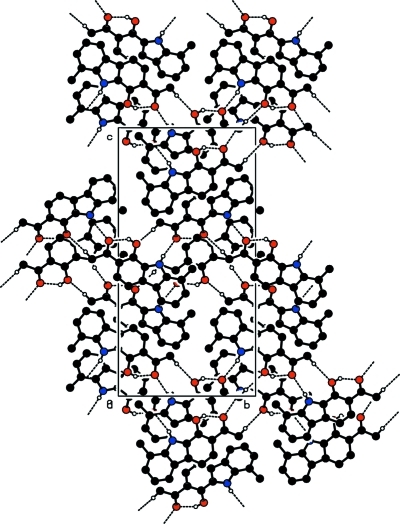
The molecular packing of the title compound, viewed down the a axis. Dashed lines indicate hydrogen bonds. H atoms not involved in hydrogen bonding have been omitted.

## supplementary crystallographic information

### Comment

The isolation of carbazole alkaloids became an active area of study since these compounds possess high levels of biological and pharmacological activity (Hagiwara *et al.*, (2000); Randelia & Patel (1982); Tovey *et al.*, (1998)). 2-acetyl-9*H*-carbazol-1-ol is a versatile intermediate to synthesis various hetero-annulated carbazole derivatives (Vandana & Prasad (2004); Vandana *et al.*, (2004)). The preparation of 2-acetyl-9*H*-carbazol-1-ol from 1-hydroxycarbazole with acetic acid and ZnCl_2_/POCl_3_ was tedious process and poor yield (22%). But in the case of 2,3,4,9-tetrahydrocarbazol-1-one with ethyl acetate in the presence of NaH/KH furnished moderate yield (38%).

The title compound, C_15_H_13_NO_2_, crystallizes in the monoclinic space group *P*2_1_, with four independent molecules (A,B,*C* and D) in the asymmetric unit. The carbazole units are almost planar. In all the four molecules, there is an O—H···O hydrogen bond involving the hydroxy substituent and the carbonyl O atom of the adjacent acetyl group, forming six-membered rings. In the crystal structure, molecules A,B,*C* and D are linked *via* N—H···O and C—H···O interactions (Table 1 & Fig. 3).

### Experimental

To the suspension of 60% sodium hydride (2.4 g, 0.06 mol) in benzene (20 ml) 8-methyl-2,3,4,9-tetrahydrocarbazol-1-one (1.6 g, 0.008 mol) was added. It was heated on a water bath for 5 min. Then small amount of potassium hydride was added carefully (caution: Dry potassium hydride is highly pyrophoric since it may contain potassium metal). It was allowed to reflux. To this mixture, ethyl acetate was added drop by drop. After the addition was completed it was allowed to reflux for 2 h. The solution was cooled, neutralized with glacial acetic acid, poured into ice water and extracted with ethyl acetate. The organic layer was dried over anhydrous sodium sulfate and the solvent was removed to get a crude mass. It was purified by column chromatography over silica gel using petroleum ether as eluant (yield: 0.726 g, 38%). It was crystallized from ethanol.

### Refinement

Owing to the absence of any anamalous scatterers in the molecule, the Friedel pairs were merged. The absolute structure in the present model have been chosen arbitrarily. H12A, H12B, H12C & H12D attached to the corresponding N atoms were located in a difference Fourier map and refined freely. H1A, H1B, H1C & H1D attached to the corresponding O atoms were located in a difference Fourier map and refined with O—H *DFIX* (0.84 (2) Å) restraints. The remaining H atoms were positioned geometrically and allowed to ride on their parent atoms, with C—H = 0.93 and 0.96 Å for C*sp*^2^ and methyl H atoms, respectively. *U*_iso_(H) = *xU*_eq_(C), where *x* = 1.5 for methyl H atoms and 1.2 for other C-bound H atoms.

### Crystal data

**Table:**

C_15_H_13_NO_2_	*F*(000) = 1008
*M**_r_* = 239.26	*D*_x_ = 1.343 Mg m^−^^3^
Monoclinic, *P*2_1_	Melting point: 485 K
Hall symbol: P 2yb	Cu *K*α radiation, λ = 1.54184 Å
*a* = 7.4602 (2) Å	Cell parameters from 4181 reflections
*b* = 12.7353 (3) Å	θ = 5.0–72.6°
*c* = 24.9325 (5) Å	µ = 0.72 mm^−^^1^
β = 92.319 (2)°	*T* = 295 K
*V* = 2366.84 (10) Å^3^	Plate, pale yellow-orange
*Z* = 8	0.47 × 0.31 × 0.11 mm

### Data collection

**Table:**

Oxford Diffraction Xcalibur Ruby Gemini diffractometer	4770 independent reflections
Radiation source: Enhance (Cu) X-ray Source	4075 reflections with *I* > 2σ(*I*)
graphite	*R*_int_ = 0.0000
Detector resolution: 10.5081 pixels mm^-1^	θ_max_ = 72.8°, θ_min_ = 5.0°
ω scans	*h* = −8→8
Absorption correction: multi-scan (*CrysAlis PRO*; Oxford Diffraction, 2010)	*k* = 0→15
*T*_min_ = 0.746, *T*_max_ = 1.000	*l* = 0→30
4770 measured reflections	

### Refinement

**Table:**

Refinement on *F*^2^	Primary atom site location: structure-invariant direct methods
Least-squares matrix: full	Secondary atom site location: difference Fourier map
*R*[*F*^2^ > 2σ(*F*^2^)] = 0.042	Hydrogen site location: inferred from neighbouring sites
*wR*(*F*^2^) = 0.122	H atoms treated by a mixture of independent and constrained refinement
*S* = 1.05	*w* = 1/[σ^2^(*F*_o_^2^) + (0.075*P*)^2^] where *P* = (*F*_o_^2^ + 2*F*_c_^2^)/3
4770 reflections	(Δ/σ)_max_ = 0.001
685 parameters	Δρ_max_ = 0.16 e Å^−^^3^
5 restraints	Δρ_min_ = −0.18 e Å^−^^3^

### Special details

**Table:**

Geometry. Bond distances, angles *etc*. have been calculated using the rounded fractional coordinates. All su's are estimated from the variances of the (full) variance-covariance matrix. The cell e.s.d.'s are taken into account in the estimation of distances, angles and torsion angles
Refinement. Refinement of *F*^2^ against ALL reflections. The weighted *R*-factor *wR* and goodness of fit *S* are based on *F*^2^, conventional *R*-factors *R* are based on *F*, with *F* set to zero for negative *F*^2^. The threshold expression of *F*^2^ > 2σ(*F*^2^) is used only for calculating *R*-factors(gt) *etc*. and is not relevant to the choice of reflections for refinement. *R*-factors based on *F*^2^ are statistically about twice as large as those based on *F*, and *R*- factors based on ALL data will be even larger.

### Fractional atomic coordinates and isotropic or equivalent isotropic displacement parameters (Å^2^)

**Table:**

	*x*	*y*	*z*	*U*_iso_*/*U*_eq_	
O1A	0.1525 (4)	1.05674 (18)	−0.05071 (10)	0.0640 (8)	
O14A	0.1171 (4)	1.25605 (19)	−0.06053 (11)	0.0675 (9)	
N12A	0.2811 (4)	0.8882 (2)	0.01958 (12)	0.0533 (8)	
C1A	0.2302 (4)	1.0783 (3)	−0.00238 (13)	0.0508 (9)	
C2A	0.2542 (4)	1.1806 (3)	0.01736 (13)	0.0525 (9)	
C3A	0.3381 (5)	1.1961 (3)	0.06884 (14)	0.0590 (10)	
C4A	0.3951 (5)	1.1146 (3)	0.10034 (14)	0.0581 (11)	
C5A	0.3719 (4)	1.0114 (3)	0.08106 (13)	0.0511 (9)	
C6A	0.4120 (4)	0.9095 (3)	0.10274 (14)	0.0523 (9)	
C7A	0.4916 (5)	0.8752 (3)	0.15137 (15)	0.0618 (11)	
C8A	0.5134 (5)	0.7691 (3)	0.15940 (16)	0.0698 (12)	
C9A	0.4583 (5)	0.6973 (3)	0.12009 (16)	0.0665 (11)	
C10A	0.3757 (4)	0.7269 (3)	0.07168 (15)	0.0560 (10)	
C11A	0.3545 (4)	0.8356 (3)	0.06368 (14)	0.0522 (9)	
C13A	0.2899 (4)	0.9948 (2)	0.03020 (13)	0.0489 (9)	
C14A	0.1936 (5)	1.2698 (3)	−0.01600 (15)	0.0578 (10)	
C15A	0.2219 (6)	1.3809 (3)	0.00336 (18)	0.0706 (15)	
C16A	0.3108 (6)	0.6483 (3)	0.03121 (18)	0.0725 (15)	
O1B	0.9083 (4)	1.0667 (2)	0.08942 (10)	0.0659 (8)	
O14B	0.8515 (4)	1.2652 (2)	0.07931 (11)	0.0722 (9)	
N12B	0.9425 (4)	0.8947 (2)	0.16560 (13)	0.0597 (9)	
C1B	0.8865 (4)	1.0842 (2)	0.14183 (13)	0.0501 (9)	
C2B	0.8459 (4)	1.1829 (2)	0.16315 (13)	0.0507 (9)	
C3B	0.8269 (4)	1.1929 (3)	0.21987 (14)	0.0590 (10)	
C4B	0.8462 (5)	1.1094 (3)	0.25346 (15)	0.0611 (11)	
C5B	0.8831 (4)	1.0106 (3)	0.23219 (13)	0.0544 (10)	
C6B	0.9096 (4)	0.9088 (3)	0.25566 (14)	0.0593 (11)	
C7B	0.9062 (5)	0.8711 (3)	0.30863 (16)	0.0723 (12)	
C8B	0.9356 (6)	0.7660 (4)	0.31717 (19)	0.0838 (17)	
C9B	0.9663 (6)	0.6985 (3)	0.2750 (2)	0.0818 (14)	
C10B	0.9708 (5)	0.7320 (3)	0.22232 (18)	0.0702 (13)	
C11B	0.9446 (5)	0.8405 (3)	0.21344 (16)	0.0597 (11)	
C13B	0.9033 (4)	0.9982 (2)	0.17697 (13)	0.0515 (9)	
C14B	0.8236 (4)	1.2732 (3)	0.12768 (15)	0.0587 (11)	
C15B	0.7684 (7)	1.3769 (3)	0.14954 (18)	0.0799 (15)	
C16B	0.9942 (7)	0.6594 (4)	0.1777 (2)	0.0906 (17)	
O1C	0.6222 (4)	0.12255 (19)	0.39711 (11)	0.0732 (9)	
O14C	0.6010 (4)	−0.0771 (2)	0.41193 (11)	0.0732 (9)	
N12C	0.5247 (4)	0.2935 (2)	0.32494 (12)	0.0565 (9)	
C1C	0.5248 (4)	0.1036 (3)	0.35163 (13)	0.0536 (10)	
C2C	0.4680 (4)	0.0027 (3)	0.33534 (13)	0.0529 (9)	
C3C	0.3690 (4)	−0.0085 (3)	0.28552 (14)	0.0561 (10)	
C4C	0.3249 (4)	0.0744 (3)	0.25361 (14)	0.0563 (10)	
C5C	0.3806 (4)	0.1755 (3)	0.26976 (12)	0.0506 (9)	
C6C	0.3622 (4)	0.2777 (3)	0.24613 (13)	0.0525 (9)	
C7C	0.2774 (5)	0.3146 (3)	0.19935 (14)	0.0613 (11)	
C8C	0.2835 (5)	0.4202 (3)	0.18826 (15)	0.0677 (11)	
C9C	0.3741 (5)	0.4886 (3)	0.22329 (15)	0.0678 (11)	
C10C	0.4615 (5)	0.4565 (3)	0.26995 (14)	0.0596 (11)	
C11C	0.4534 (4)	0.3482 (3)	0.28116 (13)	0.0528 (9)	
C13C	0.4791 (4)	0.1886 (3)	0.31835 (13)	0.0511 (9)	
C14C	0.5118 (5)	−0.0873 (3)	0.36943 (14)	0.0593 (11)	
C15C	0.4478 (7)	−0.1955 (3)	0.3533 (2)	0.0813 (15)	
C16C	0.5571 (7)	0.5312 (3)	0.30756 (18)	0.0819 (16)	
O1D	0.3083 (4)	0.13066 (19)	0.56704 (10)	0.0687 (9)	
O14D	0.3287 (4)	−0.0698 (2)	0.57844 (11)	0.0747 (9)	
N12D	0.2317 (4)	0.2998 (2)	0.49087 (11)	0.0527 (8)	
C1D	0.2471 (4)	0.1091 (3)	0.51683 (13)	0.0516 (9)	
C2D	0.2215 (4)	0.0063 (3)	0.49717 (13)	0.0522 (10)	
C3D	0.1537 (5)	−0.0082 (3)	0.44362 (14)	0.0590 (11)	
C4D	0.1133 (5)	0.0739 (3)	0.41025 (13)	0.0602 (11)	
C5D	0.1438 (4)	0.1768 (3)	0.42924 (12)	0.0512 (9)	
C6D	0.1252 (4)	0.2783 (3)	0.40517 (13)	0.0545 (10)	
C7D	0.0669 (5)	0.3119 (3)	0.35375 (14)	0.0666 (11)	
C8D	0.0673 (6)	0.4169 (3)	0.34277 (16)	0.0739 (14)	
C9D	0.1248 (6)	0.4891 (3)	0.38213 (16)	0.0680 (11)	
C10D	0.1850 (5)	0.4605 (3)	0.43328 (15)	0.0579 (11)	
C11D	0.1813 (4)	0.3520 (3)	0.44388 (13)	0.0519 (10)	
C13D	0.2106 (4)	0.1931 (2)	0.48165 (13)	0.0494 (9)	
C14D	0.2714 (5)	−0.0830 (3)	0.53138 (15)	0.0566 (10)	
C15D	0.2613 (6)	−0.1924 (3)	0.51031 (17)	0.0701 (11)	
C16D	0.2479 (6)	0.5384 (3)	0.47376 (17)	0.0713 (15)	
H1A	0.126 (8)	1.115 (3)	−0.066 (2)	0.1089*	
H3A	0.35469	1.26430	0.08145	0.0706*	
H4A	0.44856	1.12686	0.13411	0.0696*	
H7A	0.52892	0.92311	0.17769	0.0740*	
H8A	0.56570	0.74493	0.19157	0.0837*	
H9A	0.47774	0.62626	0.12659	0.0797*	
H12A	0.237 (7)	0.860 (4)	−0.0058 (19)	0.085 (15)*	
H15A	0.17640	1.42889	−0.02357	0.1059*	
H15B	0.34773	1.39322	0.01010	0.1059*	
H15C	0.15955	1.39123	0.03588	0.1059*	
H16A	0.34390	0.57910	0.04322	0.1089*	
H16B	0.36404	0.66235	−0.00246	0.1089*	
H16C	0.18261	0.65282	0.02677	0.1089*	
H1B	0.908 (9)	1.124 (3)	0.074 (2)	0.1359*	
H3B	0.80059	1.25833	0.23407	0.0708*	
H4B	0.83500	1.11789	0.29022	0.0733*	
H7B	0.88464	0.91611	0.33703	0.0863*	
H8B	0.93476	0.73953	0.35193	0.1006*	
H9B	0.98476	0.62765	0.28243	0.0978*	
H12B	0.955 (7)	0.870 (4)	0.131 (2)	0.091 (15)*	
H15D	0.73428	1.42297	0.12042	0.1200*	
H15E	0.86694	1.40709	0.17014	0.1200*	
H15F	0.66851	1.36722	0.17212	0.1200*	
H16D	1.03231	0.59224	0.19143	0.1359*	
H16E	1.08324	0.68660	0.15464	0.1359*	
H16F	0.88240	0.65163	0.15760	0.1359*	
H1C	0.617 (9)	0.063 (2)	0.411 (2)	0.1225*	
H3C	0.33337	−0.07537	0.27451	0.0673*	
H4C	0.25890	0.06460	0.22156	0.0675*	
H7C	0.21727	0.26862	0.17587	0.0734*	
H8C	0.22664	0.44603	0.15711	0.0810*	
H9C	0.37559	0.55963	0.21469	0.0811*	
H12C	0.580 (5)	0.320 (3)	0.3536 (15)	0.051 (9)*	
H15G	0.49524	−0.21380	0.31930	0.1219*	
H15H	0.31910	−0.19616	0.35033	0.1219*	
H15I	0.48832	−0.24545	0.37995	0.1219*	
H16G	0.49078	0.53843	0.33954	0.1225*	
H16H	0.56713	0.59839	0.29053	0.1225*	
H16I	0.67480	0.50456	0.31673	0.1225*	
H1D	0.345 (7)	0.076 (3)	0.584 (2)	0.1067*	
H3D	0.13630	−0.07624	0.43086	0.0708*	
H4D	0.06650	0.06229	0.37562	0.0723*	
H7D	0.02879	0.26366	0.32772	0.0799*	
H8D	0.02901	0.44059	0.30893	0.0886*	
H9D	0.12259	0.56007	0.37343	0.0816*	
H12D	0.273 (5)	0.326 (3)	0.5207 (17)	0.065 (11)*	
H15J	0.30862	−0.23999	0.53716	0.1051*	
H15K	0.33029	−0.19761	0.47875	0.1051*	
H15L	0.13852	−0.20994	0.50146	0.1051*	
H16J	0.25639	0.60613	0.45716	0.1067*	
H16K	0.36376	0.51796	0.48837	0.1067*	
H16L	0.16453	0.54170	0.50204	0.1067*	

### Atomic displacement parameters (Å^2^)

**Table:**

	*U*^11^	*U*^22^	*U*^33^	*U*^12^	*U*^13^	*U*^23^
O1A	0.0925 (17)	0.0424 (12)	0.0558 (13)	0.0037 (12)	−0.0115 (12)	−0.0051 (10)
O14A	0.0919 (17)	0.0440 (13)	0.0659 (15)	0.0055 (12)	−0.0055 (13)	0.0048 (11)
N12A	0.0678 (16)	0.0357 (13)	0.0558 (15)	−0.0036 (12)	−0.0037 (13)	−0.0022 (12)
C1A	0.0589 (16)	0.0435 (17)	0.0501 (16)	−0.0022 (13)	0.0027 (13)	−0.0027 (13)
C2A	0.0631 (16)	0.0359 (15)	0.0588 (17)	−0.0010 (13)	0.0079 (14)	−0.0042 (14)
C3A	0.079 (2)	0.0380 (16)	0.0599 (18)	−0.0063 (15)	0.0023 (16)	−0.0087 (14)
C4A	0.073 (2)	0.0481 (18)	0.0528 (17)	−0.0078 (15)	−0.0025 (15)	−0.0088 (14)
C5A	0.0569 (16)	0.0431 (16)	0.0533 (16)	−0.0026 (13)	0.0015 (13)	−0.0009 (14)
C6A	0.0533 (15)	0.0473 (17)	0.0563 (17)	−0.0026 (13)	0.0038 (13)	−0.0022 (14)
C7A	0.0642 (18)	0.061 (2)	0.0595 (19)	−0.0020 (16)	−0.0044 (15)	0.0045 (16)
C8A	0.072 (2)	0.070 (2)	0.067 (2)	0.0025 (18)	−0.0035 (17)	0.018 (2)
C9A	0.073 (2)	0.0476 (19)	0.079 (2)	0.0039 (16)	0.0060 (18)	0.0154 (18)
C10A	0.0602 (17)	0.0414 (16)	0.0667 (19)	−0.0010 (13)	0.0070 (15)	0.0038 (14)
C11A	0.0551 (16)	0.0438 (16)	0.0580 (17)	−0.0044 (13)	0.0060 (14)	0.0028 (14)
C13A	0.0567 (16)	0.0349 (15)	0.0551 (16)	−0.0010 (12)	0.0015 (13)	−0.0044 (12)
C14A	0.0674 (18)	0.0377 (16)	0.069 (2)	0.0005 (14)	0.0101 (16)	0.0019 (15)
C15A	0.095 (3)	0.0365 (16)	0.080 (3)	0.0020 (17)	0.000 (2)	−0.0004 (17)
C16A	0.090 (3)	0.0424 (18)	0.085 (3)	−0.0042 (17)	0.002 (2)	−0.0031 (17)
O1B	0.0975 (17)	0.0459 (13)	0.0545 (13)	0.0059 (12)	0.0058 (12)	0.0019 (11)
O14B	0.1069 (19)	0.0479 (14)	0.0619 (15)	0.0055 (13)	0.0038 (14)	0.0116 (12)
N12B	0.0711 (16)	0.0403 (14)	0.0672 (18)	0.0040 (12)	−0.0050 (14)	0.0053 (13)
C1B	0.0559 (16)	0.0400 (16)	0.0539 (16)	−0.0020 (12)	−0.0024 (13)	0.0037 (13)
C2B	0.0569 (16)	0.0369 (14)	0.0579 (16)	−0.0031 (12)	−0.0019 (13)	0.0026 (13)
C3B	0.0662 (18)	0.0492 (17)	0.0616 (18)	0.0019 (15)	0.0016 (15)	−0.0046 (15)
C4B	0.0716 (19)	0.0554 (19)	0.0562 (18)	−0.0022 (16)	0.0005 (15)	0.0024 (15)
C5B	0.0555 (16)	0.0513 (17)	0.0559 (17)	−0.0055 (14)	−0.0041 (13)	0.0049 (14)
C6B	0.0583 (17)	0.0557 (19)	0.063 (2)	−0.0048 (15)	−0.0087 (14)	0.0137 (16)
C7B	0.074 (2)	0.074 (2)	0.068 (2)	−0.0086 (19)	−0.0062 (18)	0.0169 (19)
C8B	0.096 (3)	0.076 (3)	0.078 (3)	−0.007 (2)	−0.012 (2)	0.035 (2)
C9B	0.087 (2)	0.058 (2)	0.098 (3)	−0.0015 (19)	−0.024 (2)	0.033 (2)
C10B	0.072 (2)	0.0476 (18)	0.089 (3)	0.0035 (16)	−0.0194 (19)	0.0149 (19)
C11B	0.0578 (17)	0.0480 (18)	0.072 (2)	−0.0015 (14)	−0.0118 (15)	0.0138 (16)
C13B	0.0530 (15)	0.0395 (15)	0.0613 (17)	−0.0038 (12)	−0.0072 (13)	0.0028 (13)
C14B	0.0619 (18)	0.0439 (17)	0.070 (2)	−0.0022 (14)	−0.0014 (15)	0.0033 (15)
C15B	0.115 (3)	0.0445 (19)	0.081 (3)	0.012 (2)	0.014 (2)	0.0106 (18)
C16B	0.110 (3)	0.062 (3)	0.099 (3)	0.022 (2)	−0.004 (3)	0.014 (2)
O1C	0.112 (2)	0.0433 (13)	0.0614 (15)	−0.0050 (13)	−0.0314 (14)	0.0027 (11)
O14C	0.1079 (19)	0.0457 (14)	0.0645 (15)	0.0020 (13)	−0.0148 (14)	0.0041 (12)
N12C	0.0740 (17)	0.0421 (14)	0.0523 (15)	−0.0021 (12)	−0.0112 (13)	−0.0014 (12)
C1C	0.0671 (18)	0.0423 (16)	0.0506 (16)	0.0012 (14)	−0.0081 (14)	−0.0005 (13)
C2C	0.0637 (17)	0.0391 (15)	0.0558 (17)	−0.0013 (13)	0.0004 (14)	−0.0009 (13)
C3C	0.0660 (18)	0.0427 (16)	0.0590 (17)	−0.0055 (14)	−0.0050 (14)	−0.0075 (14)
C4C	0.0606 (17)	0.0520 (19)	0.0553 (17)	−0.0046 (14)	−0.0082 (14)	−0.0069 (14)
C5C	0.0527 (15)	0.0473 (16)	0.0513 (15)	0.0036 (13)	−0.0029 (12)	0.0002 (14)
C6C	0.0574 (16)	0.0464 (16)	0.0534 (16)	0.0048 (13)	0.0003 (13)	0.0005 (13)
C7C	0.0684 (19)	0.060 (2)	0.0549 (17)	0.0084 (16)	−0.0056 (15)	0.0016 (16)
C8C	0.078 (2)	0.067 (2)	0.0572 (19)	0.0114 (18)	−0.0072 (16)	0.0076 (17)
C9C	0.089 (2)	0.0486 (19)	0.066 (2)	0.0113 (17)	0.0049 (18)	0.0114 (16)
C10C	0.076 (2)	0.0431 (17)	0.0596 (18)	0.0022 (15)	0.0000 (16)	0.0030 (14)
C11C	0.0625 (17)	0.0451 (16)	0.0507 (16)	0.0021 (13)	0.0004 (14)	−0.0005 (13)
C13C	0.0589 (16)	0.0415 (15)	0.0525 (16)	−0.0026 (13)	−0.0043 (13)	−0.0023 (13)
C14C	0.077 (2)	0.0389 (16)	0.0619 (19)	0.0022 (15)	0.0020 (16)	0.0004 (14)
C15C	0.105 (3)	0.0430 (19)	0.094 (3)	−0.0040 (19)	−0.020 (2)	0.0043 (19)
C16C	0.115 (3)	0.045 (2)	0.084 (3)	−0.010 (2)	−0.015 (2)	0.0025 (19)
O1D	0.1049 (19)	0.0458 (13)	0.0533 (13)	−0.0008 (12)	−0.0209 (12)	0.0007 (11)
O14D	0.1045 (19)	0.0544 (15)	0.0636 (15)	0.0026 (14)	−0.0161 (14)	0.0079 (12)
N12D	0.0693 (16)	0.0384 (13)	0.0497 (14)	0.0044 (11)	−0.0058 (12)	0.0004 (11)
C1D	0.0621 (16)	0.0423 (16)	0.0498 (16)	0.0015 (14)	−0.0055 (13)	0.0004 (13)
C2D	0.0629 (17)	0.0400 (16)	0.0536 (17)	0.0048 (13)	0.0008 (14)	0.0001 (13)
C3D	0.076 (2)	0.0411 (16)	0.0593 (19)	0.0040 (15)	−0.0055 (16)	−0.0090 (14)
C4D	0.079 (2)	0.0535 (19)	0.0475 (16)	0.0047 (16)	−0.0050 (15)	−0.0059 (14)
C5D	0.0627 (16)	0.0461 (17)	0.0445 (14)	0.0053 (14)	−0.0011 (12)	0.0020 (13)
C6D	0.0656 (18)	0.0458 (17)	0.0522 (16)	0.0069 (14)	0.0043 (14)	−0.0008 (14)
C7D	0.091 (2)	0.058 (2)	0.0502 (17)	0.0066 (18)	−0.0041 (16)	0.0043 (16)
C8D	0.095 (3)	0.065 (2)	0.061 (2)	0.012 (2)	−0.0045 (19)	0.0141 (18)
C9D	0.090 (2)	0.0450 (18)	0.069 (2)	0.0080 (17)	0.0036 (19)	0.0141 (16)
C10D	0.0672 (19)	0.0424 (17)	0.0641 (19)	0.0039 (14)	0.0025 (15)	0.0056 (14)
C11D	0.0597 (17)	0.0443 (17)	0.0516 (17)	0.0050 (13)	0.0020 (13)	0.0028 (13)
C13D	0.0580 (15)	0.0395 (15)	0.0506 (15)	0.0021 (13)	0.0004 (12)	−0.0021 (13)
C14D	0.0645 (17)	0.0412 (17)	0.064 (2)	0.0016 (14)	0.0003 (15)	0.0054 (14)
C15D	0.091 (2)	0.0459 (19)	0.073 (2)	0.0061 (17)	−0.0031 (19)	0.0020 (17)
C16D	0.089 (3)	0.0414 (18)	0.083 (3)	−0.0008 (17)	−0.004 (2)	0.0021 (17)

### Geometric parameters (Å, °)

**Table:**

O1A—C1A	1.344 (4)	C14B—C15B	1.493 (6)
O14A—C14A	1.240 (5)	C3B—H3B	0.9300
O1A—H1A	0.85 (4)	C4B—H4B	0.9300
O1B—C1B	1.342 (4)	C7B—H7B	0.9300
O14B—C14B	1.236 (5)	C8B—H8B	0.9300
O1B—H1B	0.83 (4)	C9B—H9B	0.9300
O1C—C1C	1.344 (4)	C15B—H15E	0.9600
O14C—C14C	1.235 (5)	C15B—H15F	0.9600
O1C—H1C	0.84 (3)	C15B—H15D	0.9600
O1D—C1D	1.343 (4)	C16B—H16E	0.9600
O14D—C14D	1.244 (5)	C16B—H16F	0.9600
O1D—H1D	0.85 (4)	C16B—H16D	0.9600
N12A—C11A	1.382 (5)	C1C—C2C	1.408 (5)
N12A—C13A	1.384 (4)	C1C—C13C	1.398 (5)
N12A—H12A	0.79 (5)	C2C—C3C	1.426 (5)
N12B—C13B	1.382 (4)	C2C—C14C	1.456 (5)
N12B—C11B	1.378 (5)	C3C—C4C	1.355 (5)
N12B—H12B	0.93 (5)	C4C—C5C	1.407 (5)
N12C—C11C	1.383 (4)	C5C—C6C	1.433 (5)
N12C—C13C	1.387 (5)	C5C—C13C	1.401 (4)
N12C—H12C	0.88 (4)	C6C—C11C	1.409 (5)
N12D—C13D	1.386 (4)	C6C—C7C	1.386 (5)
N12D—C11D	1.386 (4)	C7C—C8C	1.374 (5)
N12D—H12D	0.86 (4)	C8C—C9C	1.390 (5)
C1A—C13A	1.400 (5)	C9C—C10C	1.373 (5)
C1A—C2A	1.402 (5)	C10C—C11C	1.409 (5)
C2A—C14A	1.468 (5)	C10C—C16C	1.496 (6)
C2A—C3A	1.419 (5)	C14C—C15C	1.508 (6)
C3A—C4A	1.359 (5)	C3C—H3C	0.9300
C4A—C5A	1.408 (5)	C4C—H4C	0.9300
C5A—C13A	1.402 (5)	C7C—H7C	0.9300
C5A—C6A	1.433 (5)	C8C—H8C	0.9300
C6A—C11A	1.409 (5)	C9C—H9C	0.9300
C6A—C7A	1.398 (5)	C15C—H15I	0.9600
C7A—C8A	1.375 (5)	C15C—H15G	0.9600
C8A—C9A	1.390 (6)	C15C—H15H	0.9600
C9A—C10A	1.385 (5)	C16C—H16G	0.9600
C10A—C16A	1.488 (6)	C16C—H16H	0.9600
C10A—C11A	1.407 (5)	C16C—H16I	0.9600
C14A—C15A	1.507 (5)	C1D—C2D	1.408 (5)
C3A—H3A	0.9300	C1D—C13D	1.403 (5)
C4A—H4A	0.9300	C2D—C3D	1.421 (5)
C7A—H7A	0.9300	C2D—C14D	1.461 (5)
C8A—H8A	0.9300	C3D—C4D	1.362 (5)
C9A—H9A	0.9300	C4D—C5D	1.409 (5)
C15A—H15A	0.9600	C5D—C6D	1.430 (5)
C15A—H15B	0.9600	C5D—C13D	1.395 (4)
C15A—H15C	0.9600	C6D—C7D	1.404 (5)
C16A—H16A	0.9600	C6D—C11D	1.398 (5)
C16A—H16C	0.9600	C7D—C8D	1.365 (5)
C16A—H16B	0.9600	C8D—C9D	1.399 (6)
C1B—C2B	1.403 (4)	C9D—C10D	1.384 (5)
C1B—C13B	1.405 (4)	C10D—C11D	1.407 (5)
C2B—C14B	1.456 (5)	C10D—C16D	1.478 (6)
C2B—C3B	1.433 (5)	C14D—C15D	1.490 (5)
C3B—C4B	1.358 (5)	C3D—H3D	0.9300
C4B—C5B	1.397 (5)	C4D—H4D	0.9300
C5B—C13B	1.400 (5)	C7D—H7D	0.9300
C5B—C6B	1.433 (5)	C8D—H8D	0.9300
C6B—C11B	1.398 (5)	C9D—H9D	0.9300
C6B—C7B	1.406 (5)	C15D—H15J	0.9600
C7B—C8B	1.372 (6)	C15D—H15K	0.9600
C8B—C9B	1.384 (7)	C15D—H15L	0.9600
C9B—C10B	1.383 (7)	C16D—H16J	0.9600
C10B—C16B	1.462 (7)	C16D—H16K	0.9600
C10B—C11B	1.412 (5)	C16D—H16L	0.9600
			
O1A···O14A	2.563 (3)	C14C···H7B^vii^	2.9300
O1A···N12A	2.909 (4)	C14D···H16K^iv^	3.0700
O1B···C13A^i^	3.386 (4)	C14D···H1D	2.46 (4)
O1B···O14B	2.574 (4)	C15A···H16A^x^	2.8500
O1B···N12B	2.904 (4)	C15A···H3A	2.6100
O1B···C1A^i^	3.387 (4)	C15B···H3B	2.6000
O1C···N12C	2.898 (4)	C15C···H3C	2.6100
O1C···C15D^ii^	3.388 (5)	C15D···H16J^vii^	2.8900
O1C···O14C	2.575 (4)	C15D···H3D	2.6100
O1D···O14D	2.573 (4)	C16A···H12A	2.90 (5)
O1D···N12D	2.913 (4)	C16A···H1B^v^	3.05 (5)
O14A···C16B^iii^	3.247 (6)	C16B···H1A^v^	2.95 (5)
O14A···N12B^iii^	3.176 (4)	C16B···H12B	2.93 (5)
O14A···O1A	2.563 (3)	C16C···H12C	2.93 (4)
O14B···O1B	2.574 (4)	C16C···H1D^ii^	2.83 (5)
O14B···N12A^iii^	3.052 (4)	C16D···H1C^ii^	3.02 (5)
O14B···C16A^iii^	3.317 (5)	C16D···H12D	2.95 (4)
O14C···N12D^iv^	3.105 (4)	H1A···H16F^iii^	2.3300
O14C···C16D^iv^	3.361 (5)	H1A···H16C^vi^	2.5800
O14C···O1C	2.575 (4)	H1A···O14A	1.80 (4)
O14D···C16C^iv^	3.204 (5)	H1A···C16B^iii^	2.95 (5)
O14D···N12C^iv^	3.133 (4)	H1A···C14A	2.38 (4)
O14D···O1D	2.573 (4)	H1B···C16A^iii^	3.05 (6)
O1A···H12A	2.81 (5)	H1B···C14B	2.42 (5)
O1A···H15D^v^	2.6000	H1B···C2A^i^	3.08 (6)
O1A···H16C^vi^	2.8700	H1B···O14B	1.85 (4)
O1B···H15A^v^	2.4700	H1C···H16K^iv^	2.5700
O1B···H12B	2.73 (5)	H1C···C16D^iv^	3.02 (5)
O1C···H15J^ii^	2.4400	H1C···O14C	1.79 (3)
O1C···H12C	2.75 (4)	H1C···C14C	2.30 (4)
O1D···H15I^ii^	2.5300	H1D···H16G^iv^	2.2700
O1D···H12D	2.75 (4)	H1D···O14D	1.87 (4)
O14A···H1A	1.80 (4)	H1D···C14D	2.46 (4)
O14A···H16F^iii^	2.7600	H1D···C16C^iv^	2.83 (5)
O14A···H12B^iii^	2.33 (5)	H3A···C7C^x^	3.0900
O14A···H16E^iii^	2.8700	H3A···H15C	2.4300
O14A···H16C^vi^	2.7500	H3A···C15A	2.6100
O14B···H12A^iii^	2.27 (5)	H3A···H15B	2.4200
O14B···H16B^iii^	2.7800	H3B···H15E	2.5400
O14B···H1B	1.85 (4)	H3B···C15B	2.6000
O14C···H7B^vii^	2.8800	H3B···H15F	2.2700
O14C···H12D^iv^	2.26 (4)	H3C···H15H	2.4400
O14C···H1C	1.79 (3)	H3C···H15G	2.3900
O14C···H16K^iv^	2.7700	H3C···C15C	2.6100
O14D···H16G^iv^	2.7700	H3D···C15D	2.6100
O14D···H1D	1.87 (4)	H3D···H15K	2.4000
O14D···H12C^iv^	2.28 (4)	H3D···H15L	2.4500
O14D···H16I^iv^	2.7800	H4B···H7D^xi^	2.5100
N12A···O14B^v^	3.052 (4)	H4B···C1C^x^	2.8300
N12A···O1A	2.909 (4)	H4B···C13C^x^	2.9200
N12B···C7A	3.377 (5)	H4C···C5B^xii^	2.9100
N12B···O14A^v^	3.176 (4)	H4C···C13B^xii^	2.9600
N12B···O1B	2.904 (4)	H4D···H7B^xii^	2.4800
N12C···O1C	2.898 (4)	H7A···C13B	2.9500
N12C···O14D^ii^	3.133 (4)	H7B···C14C^x^	2.9300
N12D···O14C^ii^	3.105 (4)	H7B···H4D^xi^	2.4800
N12D···O1D	2.913 (4)	H7B···O14C^x^	2.8800
N12A···H15B^v^	2.9000	H7C···C3A^vii^	3.0000
N12B···H16E	2.8700	H7C···C4A^vii^	3.0600
N12C···H16I	2.9200	H7C···C2B^xii^	2.9800
N12D···H16K	2.9500	H7D···H4B^xii^	2.5100
N12D···H15L^viii^	2.7800	H7D···C4B^xii^	2.9900
C1A···O1B^ix^	3.387 (4)	H8A···C10B	3.0900
C1C···C4D	3.474 (5)	H8C···H16D^ix^	2.5300
C1C···C4B^vii^	3.496 (5)	H8D···H9B^ix^	2.4900
C2A···C16A^iii^	3.533 (5)	H9A···H16A	2.3500
C2C···C4D	3.422 (5)	H9A···H9C	2.5000
C3D···C14C	3.460 (5)	H9B···H16D	2.3500
C4B···C13C^x^	3.390 (5)	H9B···H8D^i^	2.4900
C4B···C4C^xi^	3.599 (5)	H9B···H9D^i^	2.6000
C4B···C1C^x^	3.496 (5)	H9C···H9A	2.5000
C4C···C4B^xii^	3.599 (5)	H9C···H16H	2.3800
C4C···C5B^xii^	3.416 (4)	H9C···C9A	3.0200
C4D···C2C	3.422 (5)	H9D···H16J	2.3500
C4D···C1C	3.474 (5)	H9D···H9B^ix^	2.6000
C5B···C4C^xi^	3.416 (4)	H12A···C16A	2.90 (5)
C6C···C7D	3.567 (5)	H12A···O14B^v^	2.27 (5)
C7A···N12B	3.377 (5)	H12A···O1A	2.81 (5)
C7A···C13B	3.484 (5)	H12B···C16B	2.93 (5)
C7B···C14C^x^	3.405 (5)	H12B···H16E	2.5800
C7D···C6C	3.567 (5)	H12B···O14A^v^	2.33 (5)
C7D···C13C	3.595 (5)	H12B···O1B	2.73 (5)
C7D···C11C	3.497 (5)	H12C···O1C	2.75 (4)
C8A···C11B	3.555 (5)	H12C···O14D^ii^	2.28 (4)
C8D···C11C	3.432 (5)	H12C···C16C	2.93 (4)
C8D···C10C	3.554 (6)	H12D···C16D	2.95 (4)
C10A···C14A^v^	3.593 (5)	H12D···O1D	2.75 (4)
C10C···C8D	3.554 (6)	H12D···O14C^ii^	2.26 (4)
C10D···C14D^viii^	3.594 (5)	H15A···O1B^iii^	2.4700
C11B···C8A	3.555 (5)	H15B···H3A	2.4200
C11C···C8D	3.432 (5)	H15B···C3A	2.9100
C11C···C7D	3.497 (5)	H15B···C11A^iii^	3.0300
C11D···C15D^viii^	3.582 (5)	H15B···H16A^x^	2.5100
C11D···C14D^viii^	3.555 (5)	H15B···N12A^iii^	2.9000
C13A···O1B^ix^	3.386 (4)	H15C···C3A	2.9200
C13B···C7A	3.484 (5)	H15C···H3A	2.4300
C13C···C4B^vii^	3.390 (5)	H15D···O1A^iii^	2.6000
C13C···C7D	3.595 (5)	H15E···H3B	2.5400
C14A···C10A^iii^	3.593 (5)	H15E···C3B	3.0200
C14C···C3D	3.460 (5)	H15F···C9C^x^	3.0100
C14C···C7B^vii^	3.405 (5)	H15F···C3B	2.7600
C14D···C10D^xiii^	3.594 (5)	H15F···C7C^x^	3.1000
C14D···C11D^xiii^	3.555 (5)	H15F···C8C^x^	2.9900
C15A···C16A^x^	3.533 (5)	H15F···H3B	2.2700
C15D···C11D^xiii^	3.582 (5)	H15G···H3C	2.3900
C15D···O1C^iv^	3.388 (5)	H15G···C3C	2.8900
C15D···C16D^vii^	3.548 (5)	H15G···H16H^vii^	2.5600
C16A···C15A^vii^	3.533 (5)	H15H···C3C	2.9200
C16A···C2A^v^	3.533 (5)	H15H···H3C	2.4400
C16A···O14B^v^	3.317 (5)	H15H···C8B^xii^	2.9800
C16B···O14A^v^	3.247 (6)	H15I···O1D^iv^	2.5300
C16C···O14D^ii^	3.204 (5)	H15J···O1C^iv^	2.4400
C16D···O14C^ii^	3.361 (5)	H15K···C3D	2.8700
C16D···C15D^x^	3.548 (5)	H15K···H3D	2.4000
C1C···H4B^vii^	2.8300	H15L···H3D	2.4500
C2A···H1B^ix^	3.08 (6)	H15L···C3D	2.9500
C2A···H16B^iii^	2.9000	H15L···C11D^xiii^	2.9000
C2B···H7C^xi^	2.9800	H15L···N12D^xiii^	2.7800
C2D···H16L^xiii^	2.9200	H15L···C13D^xiii^	2.9300
C3A···H7C^x^	3.0000	H16A···H15B^vii^	2.5100
C3A···H15C	2.9200	H16A···C15A^vii^	2.8500
C3A···H15B	2.9100	H16A···H9A	2.3500
C3A···H16B^iii^	2.8600	H16B···C2A^v^	2.9000
C3B···H15E	3.0200	H16B···O14B^v^	2.7800
C3B···H15F	2.7600	H16B···C3A^v^	2.8600
C3C···H15H	2.9200	H16C···O1A^xiv^	2.8700
C3C···H15G	2.8900	H16C···O14A^xiv^	2.7500
C3D···H15K	2.8700	H16C···H1A^xiv^	2.5800
C3D···H16L^xiii^	2.8500	H16D···H8C^i^	2.5300
C3D···H15L	2.9500	H16D···H9B	2.3500
C4A···H7C^x^	3.0600	H16D···C9C^i^	2.9500
C4B···H7D^xi^	2.9900	H16D···C8C^i^	2.8900
C5B···H4C^xi^	2.9100	H16E···H12B	2.5800
C7C···H15F^vii^	3.1000	H16E···N12B	2.8700
C7C···H3A^vii^	3.0900	H16E···C9A^i^	2.9600
C8B···H15H^xi^	2.9800	H16E···O14A^v^	2.8700
C8C···H15F^vii^	2.9900	H16F···H1A^v^	2.3300
C8C···H16D^ix^	2.8900	H16F···O14A^v^	2.7600
C9A···H16E^ix^	2.9600	H16G···C9D	3.0400
C9A···H9C	3.0200	H16G···O14D^ii^	2.7700
C9C···H15F^vii^	3.0100	H16G···H1D^ii^	2.2700
C9C···H16D^ix^	2.9500	H16H···H9C	2.3800
C9D···H16G	3.0400	H16H···H15G^x^	2.5600
C10B···H8A	3.0900	H16I···N12C	2.9200
C11A···H15B^v^	3.0300	H16I···O14D^ii^	2.7800
C11D···H15L^viii^	2.9000	H16J···C15D^x^	2.8900
C13B···H4C^xi^	2.9600	H16J···H9D	2.3500
C13B···H7A	2.9500	H16K···N12D	2.9500
C13C···H4B^vii^	2.9200	H16K···O14C^ii^	2.7700
C13D···H15L^viii^	2.9300	H16K···C14D^ii^	3.0700
C14A···H1A	2.38 (4)	H16K···H1C^ii^	2.5700
C14B···H1B	2.42 (5)	H16L···C2D^viii^	2.9200
C14C···H1C	2.30 (4)	H16L···C3D^viii^	2.8500
			
C1A—O1A—H1A	108 (3)	H15D—C15B—H15E	110.00
C1B—O1B—H1B	108 (3)	H15E—C15B—H15F	109.00
C1C—O1C—H1C	99 (4)	H16D—C16B—H16E	109.00
C1D—O1D—H1D	113 (3)	C10B—C16B—H16E	110.00
C11A—N12A—C13A	108.0 (3)	H16D—C16B—H16F	109.00
C11A—N12A—H12A	124 (4)	C10B—C16B—H16F	110.00
C13A—N12A—H12A	128 (4)	C10B—C16B—H16D	110.00
C11B—N12B—C13B	107.2 (3)	H16E—C16B—H16F	109.00
C13B—N12B—H12B	123 (3)	C2C—C1C—C13C	118.2 (3)
C11B—N12B—H12B	130 (3)	O1C—C1C—C2C	123.6 (3)
C11C—N12C—C13C	107.9 (3)	O1C—C1C—C13C	118.2 (3)
C13C—N12C—H12C	125 (2)	C1C—C2C—C14C	119.5 (3)
C11C—N12C—H12C	127 (2)	C3C—C2C—C14C	121.7 (3)
C11D—N12D—C13D	107.8 (3)	C1C—C2C—C3C	118.8 (3)
C13D—N12D—H12D	124 (3)	C2C—C3C—C4C	122.6 (3)
C11D—N12D—H12D	128 (3)	C3C—C4C—C5C	118.9 (3)
O1A—C1A—C2A	123.3 (3)	C4C—C5C—C13C	119.7 (3)
C2A—C1A—C13A	118.0 (3)	C6C—C5C—C13C	106.6 (3)
O1A—C1A—C13A	118.7 (3)	C4C—C5C—C6C	133.7 (3)
C1A—C2A—C3A	119.5 (3)	C5C—C6C—C11C	106.8 (3)
C3A—C2A—C14A	121.3 (3)	C7C—C6C—C11C	119.9 (3)
C1A—C2A—C14A	119.2 (3)	C5C—C6C—C7C	133.4 (3)
C2A—C3A—C4A	122.2 (3)	C6C—C7C—C8C	118.9 (3)
C3A—C4A—C5A	119.0 (3)	C7C—C8C—C9C	120.4 (3)
C4A—C5A—C6A	134.1 (3)	C8C—C9C—C10C	123.3 (4)
C6A—C5A—C13A	106.4 (3)	C11C—C10C—C16C	121.4 (3)
C4A—C5A—C13A	119.5 (3)	C9C—C10C—C11C	115.9 (3)
C7A—C6A—C11A	119.8 (3)	C9C—C10C—C16C	122.7 (3)
C5A—C6A—C11A	106.9 (3)	N12C—C11C—C6C	109.2 (3)
C5A—C6A—C7A	133.2 (3)	C6C—C11C—C10C	121.6 (3)
C6A—C7A—C8A	118.5 (4)	N12C—C11C—C10C	129.2 (3)
C7A—C8A—C9A	120.9 (4)	N12C—C13C—C1C	128.5 (3)
C8A—C9A—C10A	122.9 (4)	C1C—C13C—C5C	121.9 (3)
C11A—C10A—C16A	122.3 (3)	N12C—C13C—C5C	109.6 (3)
C9A—C10A—C11A	115.8 (3)	C2C—C14C—C15C	120.2 (3)
C9A—C10A—C16A	121.9 (3)	O14C—C14C—C2C	121.2 (3)
C6A—C11A—C10A	122.0 (3)	O14C—C14C—C15C	118.6 (3)
N12A—C11A—C6A	109.0 (3)	C2C—C3C—H3C	119.00
N12A—C11A—C10A	129.0 (3)	C4C—C3C—H3C	119.00
C1A—C13A—C5A	121.8 (3)	C5C—C4C—H4C	121.00
N12A—C13A—C5A	109.7 (3)	C3C—C4C—H4C	121.00
N12A—C13A—C1A	128.5 (3)	C6C—C7C—H7C	121.00
C2A—C14A—C15A	120.6 (3)	C8C—C7C—H7C	121.00
O14A—C14A—C2A	121.2 (3)	C7C—C8C—H8C	120.00
O14A—C14A—C15A	118.2 (3)	C9C—C8C—H8C	120.00
C2A—C3A—H3A	119.00	C8C—C9C—H9C	118.00
C4A—C3A—H3A	119.00	C10C—C9C—H9C	118.00
C5A—C4A—H4A	121.00	C14C—C15C—H15H	109.00
C3A—C4A—H4A	120.00	C14C—C15C—H15I	109.00
C6A—C7A—H7A	121.00	H15G—C15C—H15H	109.00
C8A—C7A—H7A	121.00	C14C—C15C—H15G	109.00
C7A—C8A—H8A	120.00	H15G—C15C—H15I	109.00
C9A—C8A—H8A	119.00	H15H—C15C—H15I	109.00
C10A—C9A—H9A	119.00	H16G—C16C—H16I	109.00
C8A—C9A—H9A	119.00	C10C—C16C—H16I	109.00
C14A—C15A—H15C	109.00	H16G—C16C—H16H	110.00
H15B—C15A—H15C	109.00	C10C—C16C—H16G	109.00
C14A—C15A—H15B	109.00	C10C—C16C—H16H	109.00
C14A—C15A—H15A	109.00	H16H—C16C—H16I	109.00
H15A—C15A—H15C	110.00	C2D—C1D—C13D	118.1 (3)
H15A—C15A—H15B	110.00	O1D—C1D—C2D	123.4 (3)
H16A—C16A—H16B	109.00	O1D—C1D—C13D	118.5 (3)
C10A—C16A—H16C	109.00	C1D—C2D—C3D	119.1 (3)
C10A—C16A—H16B	109.00	C1D—C2D—C14D	119.5 (3)
H16A—C16A—H16C	109.00	C3D—C2D—C14D	121.4 (3)
H16B—C16A—H16C	109.00	C2D—C3D—C4D	122.4 (3)
C10A—C16A—H16A	109.00	C3D—C4D—C5D	118.7 (3)
C2B—C1B—C13B	118.5 (3)	C6D—C5D—C13D	106.5 (3)
O1B—C1B—C13B	117.8 (3)	C4D—C5D—C6D	133.5 (3)
O1B—C1B—C2B	123.7 (3)	C4D—C5D—C13D	120.0 (3)
C1B—C2B—C3B	119.0 (3)	C5D—C6D—C7D	132.9 (3)
C3B—C2B—C14B	121.1 (3)	C5D—C6D—C11D	107.2 (3)
C1B—C2B—C14B	119.9 (3)	C7D—C6D—C11D	119.9 (3)
C2B—C3B—C4B	121.8 (3)	C6D—C7D—C8D	118.6 (3)
C3B—C4B—C5B	119.3 (3)	C7D—C8D—C9D	120.4 (4)
C6B—C5B—C13B	106.3 (3)	C8D—C9D—C10D	123.5 (4)
C4B—C5B—C13B	120.4 (3)	C9D—C10D—C16D	122.3 (3)
C4B—C5B—C6B	133.3 (3)	C9D—C10D—C11D	115.1 (3)
C5B—C6B—C11B	106.4 (3)	C11D—C10D—C16D	122.6 (3)
C5B—C6B—C7B	133.2 (3)	N12D—C11D—C6D	109.0 (3)
C7B—C6B—C11B	120.4 (3)	N12D—C11D—C10D	128.5 (3)
C6B—C7B—C8B	118.0 (4)	C6D—C11D—C10D	122.5 (3)
C7B—C8B—C9B	121.2 (4)	C1D—C13D—C5D	121.7 (3)
C8B—C9B—C10B	122.8 (4)	N12D—C13D—C1D	128.8 (3)
C11B—C10B—C16B	121.3 (4)	N12D—C13D—C5D	109.6 (3)
C9B—C10B—C11B	116.3 (4)	O14D—C14D—C2D	121.0 (3)
C9B—C10B—C16B	122.4 (4)	O14D—C14D—C15D	118.0 (3)
C6B—C11B—C10B	121.3 (4)	C2D—C14D—C15D	120.9 (3)
N12B—C11B—C10B	128.5 (4)	C2D—C3D—H3D	119.00
N12B—C11B—C6B	110.1 (3)	C4D—C3D—H3D	119.00
N12B—C13B—C5B	110.0 (3)	C3D—C4D—H4D	121.00
N12B—C13B—C1B	129.0 (3)	C5D—C4D—H4D	121.00
C1B—C13B—C5B	121.0 (3)	C6D—C7D—H7D	121.00
C2B—C14B—C15B	120.2 (3)	C8D—C7D—H7D	121.00
O14B—C14B—C15B	119.3 (3)	C7D—C8D—H8D	120.00
O14B—C14B—C2B	120.5 (3)	C9D—C8D—H8D	120.00
C2B—C3B—H3B	119.00	C8D—C9D—H9D	118.00
C4B—C3B—H3B	119.00	C10D—C9D—H9D	118.00
C3B—C4B—H4B	120.00	C14D—C15D—H15J	109.00
C5B—C4B—H4B	120.00	C14D—C15D—H15K	109.00
C8B—C7B—H7B	121.00	C14D—C15D—H15L	109.00
C6B—C7B—H7B	121.00	H15J—C15D—H15K	110.00
C7B—C8B—H8B	119.00	H15J—C15D—H15L	110.00
C9B—C8B—H8B	119.00	H15K—C15D—H15L	110.00
C10B—C9B—H9B	119.00	C10D—C16D—H16J	109.00
C8B—C9B—H9B	119.00	C10D—C16D—H16K	109.00
C14B—C15B—H15E	109.00	C10D—C16D—H16L	109.00
C14B—C15B—H15F	109.00	H16J—C16D—H16K	109.00
C14B—C15B—H15D	109.00	H16J—C16D—H16L	109.00
H15D—C15B—H15F	109.00	H16K—C16D—H16L	109.00
			
C13A—N12A—C11A—C6A	−0.4 (4)	C6B—C7B—C8B—C9B	−0.5 (6)
C13A—N12A—C11A—C10A	−179.8 (3)	C7B—C8B—C9B—C10B	0.5 (7)
C11A—N12A—C13A—C1A	−179.2 (3)	C8B—C9B—C10B—C16B	−176.8 (4)
C11A—N12A—C13A—C5A	0.9 (4)	C8B—C9B—C10B—C11B	0.9 (6)
C11B—N12B—C13B—C1B	179.8 (3)	C9B—C10B—C11B—N12B	180.0 (4)
C13B—N12B—C11B—C6B	−0.9 (4)	C16B—C10B—C11B—C6B	175.4 (4)
C13B—N12B—C11B—C10B	177.1 (4)	C16B—C10B—C11B—N12B	−2.3 (6)
C11B—N12B—C13B—C5B	0.9 (4)	C9B—C10B—C11B—C6B	−2.3 (5)
C11C—N12C—C13C—C5C	0.9 (4)	O1C—C1C—C2C—C3C	−178.4 (3)
C13C—N12C—C11C—C10C	179.1 (3)	O1C—C1C—C13C—C5C	178.9 (3)
C11C—N12C—C13C—C1C	178.4 (3)	C2C—C1C—C13C—N12C	−177.6 (3)
C13C—N12C—C11C—C6C	−0.3 (4)	C2C—C1C—C13C—C5C	−0.4 (5)
C11D—N12D—C13D—C1D	178.9 (3)	O1C—C1C—C2C—C14C	1.4 (5)
C13D—N12D—C11D—C10D	−177.9 (3)	C13C—C1C—C2C—C3C	0.9 (4)
C13D—N12D—C11D—C6D	1.1 (4)	C13C—C1C—C2C—C14C	−179.3 (3)
C11D—N12D—C13D—C5D	−1.1 (4)	O1C—C1C—C13C—N12C	1.7 (5)
O1A—C1A—C13A—N12A	0.2 (5)	C3C—C2C—C14C—O14C	178.4 (3)
C13A—C1A—C2A—C14A	179.8 (3)	C3C—C2C—C14C—C15C	−1.8 (5)
C2A—C1A—C13A—N12A	−180.0 (3)	C1C—C2C—C3C—C4C	−1.1 (5)
C2A—C1A—C13A—C5A	−0.2 (5)	C14C—C2C—C3C—C4C	179.1 (3)
O1A—C1A—C13A—C5A	−179.9 (3)	C1C—C2C—C14C—O14C	−1.4 (5)
C13A—C1A—C2A—C3A	0.4 (5)	C1C—C2C—C14C—C15C	178.4 (3)
O1A—C1A—C2A—C3A	−179.8 (3)	C2C—C3C—C4C—C5C	0.8 (5)
O1A—C1A—C2A—C14A	−0.5 (5)	C3C—C4C—C5C—C6C	178.3 (3)
C3A—C2A—C14A—O14A	−178.6 (3)	C3C—C4C—C5C—C13C	−0.4 (5)
C1A—C2A—C3A—C4A	−0.7 (5)	C4C—C5C—C13C—N12C	177.8 (3)
C14A—C2A—C3A—C4A	180.0 (3)	C4C—C5C—C13C—C1C	0.2 (5)
C1A—C2A—C14A—O14A	2.1 (5)	C6C—C5C—C13C—N12C	−1.2 (4)
C3A—C2A—C14A—C15A	1.0 (5)	C4C—C5C—C6C—C7C	2.5 (6)
C1A—C2A—C14A—C15A	−178.3 (3)	C4C—C5C—C6C—C11C	−177.8 (3)
C2A—C3A—C4A—C5A	0.7 (5)	C13C—C5C—C6C—C7C	−178.7 (4)
C3A—C4A—C5A—C6A	−179.0 (4)	C13C—C5C—C6C—C11C	1.0 (3)
C3A—C4A—C5A—C13A	−0.4 (5)	C6C—C5C—C13C—C1C	−178.8 (3)
C6A—C5A—C13A—N12A	−1.0 (3)	C5C—C6C—C11C—C10C	−179.9 (3)
C13A—C5A—C6A—C7A	−179.6 (4)	C7C—C6C—C11C—C10C	−0.1 (5)
C13A—C5A—C6A—C11A	0.8 (3)	C11C—C6C—C7C—C8C	−0.4 (5)
C4A—C5A—C6A—C7A	−0.8 (6)	C7C—C6C—C11C—N12C	179.3 (3)
C4A—C5A—C6A—C11A	179.5 (4)	C5C—C6C—C7C—C8C	179.4 (3)
C4A—C5A—C13A—C1A	0.2 (5)	C5C—C6C—C11C—N12C	−0.4 (4)
C4A—C5A—C13A—N12A	−180.0 (3)	C6C—C7C—C8C—C9C	0.3 (6)
C6A—C5A—C13A—C1A	179.1 (3)	C7C—C8C—C9C—C10C	0.1 (6)
C5A—C6A—C11A—C10A	179.2 (3)	C8C—C9C—C10C—C11C	−0.6 (5)
C5A—C6A—C11A—N12A	−0.2 (4)	C8C—C9C—C10C—C16C	−179.4 (4)
C7A—C6A—C11A—N12A	−180.0 (3)	C9C—C10C—C11C—C6C	0.5 (5)
C7A—C6A—C11A—C10A	−0.5 (5)	C16C—C10C—C11C—N12C	0.1 (6)
C11A—C6A—C7A—C8A	0.7 (5)	C16C—C10C—C11C—C6C	179.4 (3)
C5A—C6A—C7A—C8A	−178.9 (3)	C9C—C10C—C11C—N12C	−178.8 (3)
C6A—C7A—C8A—C9A	0.2 (6)	O1D—C1D—C2D—C3D	179.1 (3)
C7A—C8A—C9A—C10A	−1.5 (6)	O1D—C1D—C2D—C14D	−3.2 (5)
C8A—C9A—C10A—C11A	1.7 (5)	C13D—C1D—C2D—C3D	−2.4 (4)
C8A—C9A—C10A—C16A	−177.3 (4)	C13D—C1D—C2D—C14D	175.3 (3)
C9A—C10A—C11A—N12A	178.7 (3)	O1D—C1D—C13D—N12D	1.2 (5)
C9A—C10A—C11A—C6A	−0.7 (5)	O1D—C1D—C13D—C5D	−178.9 (3)
C16A—C10A—C11A—C6A	178.3 (3)	C2D—C1D—C13D—N12D	−177.3 (3)
C16A—C10A—C11A—N12A	−2.4 (5)	C2D—C1D—C13D—C5D	2.6 (4)
O1B—C1B—C2B—C3B	−179.5 (3)	C1D—C2D—C3D—C4D	0.4 (5)
C13B—C1B—C2B—C3B	1.3 (4)	C14D—C2D—C3D—C4D	−177.2 (3)
C13B—C1B—C2B—C14B	−178.6 (3)	C1D—C2D—C14D—O14D	3.2 (5)
O1B—C1B—C2B—C14B	0.6 (5)	C1D—C2D—C14D—C15D	−174.6 (3)
O1B—C1B—C13B—N12B	0.9 (5)	C3D—C2D—C14D—O14D	−179.2 (3)
O1B—C1B—C13B—C5B	179.7 (3)	C3D—C2D—C14D—C15D	3.1 (5)
C2B—C1B—C13B—N12B	−179.8 (3)	C2D—C3D—C4D—C5D	1.5 (5)
C2B—C1B—C13B—C5B	−1.1 (4)	C3D—C4D—C5D—C6D	176.7 (3)
C3B—C2B—C14B—O14B	175.4 (3)	C3D—C4D—C5D—C13D	−1.3 (5)
C1B—C2B—C3B—C4B	−0.4 (5)	C4D—C5D—C6D—C7D	1.1 (6)
C14B—C2B—C3B—C4B	179.6 (3)	C4D—C5D—C6D—C11D	−178.3 (3)
C3B—C2B—C14B—C15B	−4.0 (5)	C13D—C5D—C6D—C7D	179.3 (3)
C1B—C2B—C14B—C15B	175.9 (3)	C13D—C5D—C6D—C11D	0.0 (3)
C1B—C2B—C14B—O14B	−4.7 (5)	C4D—C5D—C13D—N12D	179.2 (3)
C2B—C3B—C4B—C5B	−0.9 (5)	C4D—C5D—C13D—C1D	−0.7 (5)
C3B—C4B—C5B—C13B	1.2 (5)	C6D—C5D—C13D—N12D	0.7 (3)
C3B—C4B—C5B—C6B	−179.6 (3)	C6D—C5D—C13D—C1D	−179.3 (3)
C4B—C5B—C6B—C11B	−179.2 (4)	C5D—C6D—C7D—C8D	−179.0 (4)
C6B—C5B—C13B—N12B	−0.6 (3)	C11D—C6D—C7D—C8D	0.3 (5)
C4B—C5B—C6B—C7B	0.8 (6)	C5D—C6D—C11D—N12D	−0.6 (3)
C4B—C5B—C13B—N12B	178.8 (3)	C5D—C6D—C11D—C10D	178.4 (3)
C13B—C5B—C6B—C7B	−179.9 (3)	C7D—C6D—C11D—N12D	179.9 (3)
C13B—C5B—C6B—C11B	0.1 (3)	C7D—C6D—C11D—C10D	−1.1 (5)
C6B—C5B—C13B—C1B	−179.6 (3)	C6D—C7D—C8D—C9D	0.0 (6)
C4B—C5B—C13B—C1B	−0.2 (5)	C7D—C8D—C9D—C10D	0.6 (7)
C5B—C6B—C11B—C10B	−177.6 (3)	C8D—C9D—C10D—C11D	−1.3 (6)
C11B—C6B—C7B—C8B	−0.9 (5)	C8D—C9D—C10D—C16D	178.9 (4)
C5B—C6B—C11B—N12B	0.5 (4)	C9D—C10D—C11D—N12D	−179.7 (3)
C7B—C6B—C11B—N12B	−179.5 (3)	C9D—C10D—C11D—C6D	1.5 (5)
C7B—C6B—C11B—C10B	2.3 (5)	C16D—C10D—C11D—N12D	0.1 (6)
C5B—C6B—C7B—C8B	179.1 (4)	C16D—C10D—C11D—C6D	−178.7 (3)

Symmetry codes: (i) *x*+1, *y*, *z*; (ii) −*x*+1, *y*+1/2, −*z*+1; (iii) −*x*+1, *y*+1/2, −*z*; (iv) −*x*+1, *y*−1/2, −*z*+1; (v) −*x*+1, *y*−1/2, −*z*; (vi) −*x*, *y*+1/2, −*z*; (vii) *x*, *y*−1, *z*; (viii) −*x*, *y*+1/2, −*z*+1; (ix) *x*−1, *y*, *z*; (x) *x*, *y*+1, *z*; (xi) *x*+1, *y*+1, *z*; (xii) *x*−1, *y*−1, *z*; (xiii) −*x*, *y*−1/2, −*z*+1; (xiv) −*x*, *y*−1/2, −*z*.

### Hydrogen-bond geometry (Å, °)

**Table:**

*D*—H···*A*	*D*—H	H···*A*	*D*···*A*	*D*—H···*A*
O1A—H1A···O14A	0.85 (4)	1.80 (4)	2.563 (3)	147 (5)
O1B—H1B···O14B	0.83 (4)	1.85 (4)	2.574 (4)	145 (5)
O1C—H1C···O14C	0.84 (3)	1.79 (3)	2.575 (4)	156 (5)
O1D—H1D···O14D	0.85 (4)	1.87 (4)	2.573 (4)	139 (5)
N12A—H12A···O14B^v^	0.79 (5)	2.27 (5)	3.052 (4)	172 (5)
N12B—H12B···O14A^v^	0.93 (5)	2.33 (5)	3.176 (4)	153 (5)
N12C—H12C···O14D^ii^	0.88 (4)	2.28 (4)	3.133 (4)	163 (3)
N12D—H12D···O14C^ii^	0.86 (4)	2.26 (4)	3.105 (4)	168 (3)
C15A—H15A···O1B^iii^	0.96	2.47	3.422 (5)	173
C15B—H15D···O1A^iii^	0.96	2.60	3.433 (5)	145
C15C—H15I···O1D^iv^	0.96	2.53	3.445 (5)	160
C15D—H15J···O1C^iv^	0.96	2.44	3.388 (5)	170

Symmetry codes: (v) −*x*+1, *y*−1/2, −*z*; (ii) −*x*+1, *y*+1/2, −*z*+1; (iii) −*x*+1, *y*+1/2, −*z*; (iv) −*x*+1, *y*−1/2, −*z*+1.
